# Pregnancy-related factors may signal additional protection or risk of future cardiovascular diseases

**DOI:** 10.1186/s12905-022-02125-x

**Published:** 2022-12-17

**Authors:** Shivani M. Reddy, Tamy H. M. Tsujimoto, Bajhat F. Qaqish, Jason P. Fine, Wanda K. Nicholson

**Affiliations:** 1 Division of Translational Health Sciences, RTI International, 307 Waverly Oaks Road, #1023, Waltham, MA 02452 USA; 2 Department of Biostatistics, Gillings School of Global Public Health, The University of North Carolina at Chapel Hill, 3105-B McGavran-Greenberg Hall, CB 7420, Chapel Hill, NC 27599-7420 USA; 3 School of Medicine, The University of North Carolina at Chapel Hill, 3027 Old Clinic Building CB#7570, Chapel Hill, USA

**Keywords:** Cardiovascular disease, Risk factors, Pregnancy complications, Breastfeeding

## Abstract

**Background:**

Cardiovascular disease (CVD) guidelines recommend using the Pooled Cohort Equation (PCE) to assess 10-year CVD risk based on traditional risk factors. Pregnancy-related factors have been associated with future CVD. We examined the contribution of two pregnancy-related factors, (1) history of a low birthweight (LBW) infant and (2) breastfeeding to CVD risk accounting for traditional risk factors as assessed by the PCE.

**Methods:**

A nationally representative sample of women, ages 40–79, with a history of pregnancy, but no prior CVD, was identified using NHANES 1999–2006. Outcomes included (1) CVD death and (2) CVD death plus CVD surrogates. We used Cox proportional hazards models to adjust for PCE risk score.

**Results:**

Among 3,758 women, 479 had a LBW infant and 1,926 reported breastfeeding. Mean follow-up time was 12.1 years. Survival models showed a consistent reduction in CVD outcomes among women with a history of breastfeeding. In cause-specific survival models, breastfeeding was associated with a 24% reduction in risk of CVD deaths (HR 0.76; 95% CI 0.45─1.27, *p* = 0.30) and a 33% reduction in risk of CVD deaths + surrogate CVD, though not statistically significant. (HR 0.77; 95% CI 0.52─1.14, *p* = 0.19). Survival models yielded inconclusive results for LBW with wide confidence intervals (CVD death: HR 0.98; 95% CI 0.47─2.05; *p* = 0.96 and CVD death + surrogate CVD: HR 1.29; 95% CI 0.74─2.25; *p* = 0.38).

**Conclusion:**

Pregnancy-related factors may provide important, relevant information about CVD risk beyond traditional risk factors. While further research with more robust datasets is needed, it may be helpful for clinicians to counsel women about the potential impact of pregnancy-related factors, particularly the positive impact of breastfeeding, on cardiovascular health.

## Background

Cardiovascular disease (CVD) is the leading cause of death in women, with an estimated prevalence of over 60 million women in the Unites States between 2013 and 2016 [1, 2]. Professional societies have adopted a risk-based approach to prevention of CVD. The American College of Cardiology (ACC) [3], the American Heart Association (AHA) [3], and the United States Preventive Services Task Force [4, 5] recommend using a risk calculator that incorporates traditional risk factors—age, blood pressure, cholesterol, tobacco use, and diabetes—to estimate the absolute 10-year risk of CVD. Lifestyle modification counseling and medications are offered to high-risk individuals.

Pregnancy-related risk factors may be associated with future CVD events and are not captured by traditional risk calculators [6]. Nearly 90 percent of US women become pregnant, averaging two children each [7], and over one-third will experience pregnancy complications [8], such as hypertensive disorders of pregnancy (gestational hypertension, pre-eclampsia), low birthweight infant, gestational diabetes, and pre-term delivery [9]. Pregnancy complications are associated with an increased relative risk of cardiovascular risk factors of hypertension [10] and diabetes, as well as CVD [11–19]. Additionally, growing evidence suggests breastfeeding is associated with short term and long-term cardiovascular benefit, including fewer markers of subclinical vascular disease in premenopausal years [20, 21]. Some guidelines recommend screening for pregnancy complications, though they offer varying details of guidance on how to modify management of patients who screen positive [6, 22, 23]. Furthermore, it remains unclear if pregnancy-related factors are independent predictors of CVD or simply markers of underlying cardiovascular risk captured by traditional risk factors.

In this study, we examine the association of two pregnancy-related factors, low birthweight (LBW) infant and breastfeeding, in a nationally representative dataset to determine if these predictors confer additional information about CVD risk beyond that of traditional risk factors. We hypothesize that women with a LBW infant have a higher risk of CVD after accounting for traditional risk factors as assessed by the ACC/AHA Pooled Cohort Equation (PCE) risk score. In contrast, we hypothesize women with a history of breastfeeding have a lower risk of CVD after accounting for the AHA/ACC PCE risk score.

## Methods

### Study participants

The study sample consisted of women surveyed by the National Health and Nutrition Examination Survey (NHANES) at one point of time between 1999 and 2006. NHANES is an annual survey that employs a complex, multistage probability sampling design to select a representative sample of the civilian noninstitutionalized U.S. population [24]. NHANES oversamples subgroups that may be underrepresented including African Americans, Mexican Americans, and low-income White Americans. NHANES is a de-identified, publicly available dataset; thus, informed consent of the participants was not required. All methods were in accordance with relevant guidelines and regulations. IRBs at RTI International and UNC have reviewed and approved this study.

**Fig. 1:**
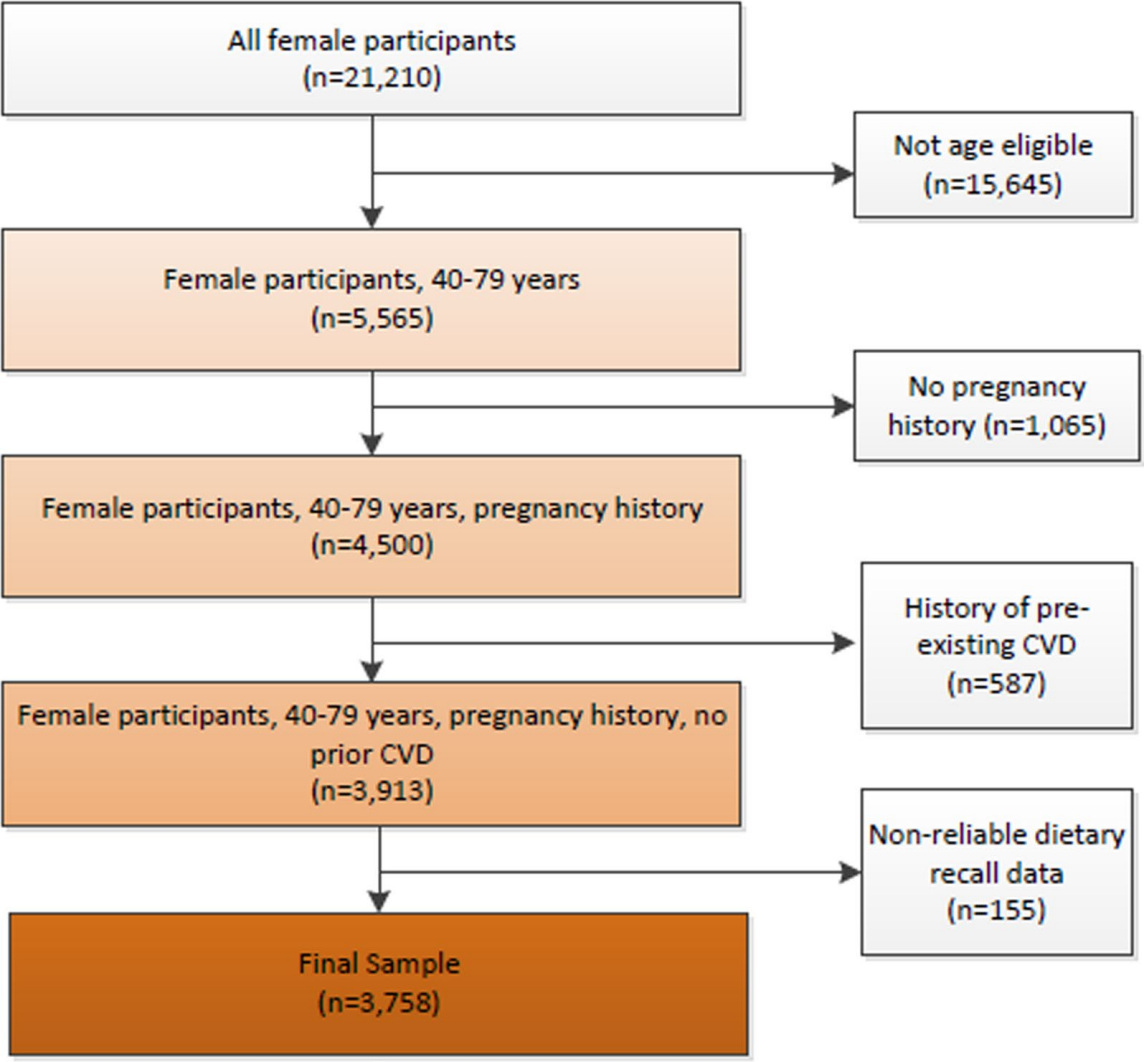
Study sample

We included women, ages 40 to 79 years, with a history of pregnancy or current pregnancy. Women younger than 40 years or older than 79 years were excluded because the ACC/AHA PCE risk calculator is not validated for use in these age groups. Women reporting pre-existing coronary artery disease, congestive heart failure, myocardial infarction, angina, or stroke were also excluded because the ACC/AHA risk calculator is used for primary prevention only. Participants who did not attend the medical examination portion of the NHANES survey and did not complete two days of dietary recall were excluded. The final sample consisted of 3758 women (Fig. 1).

### Pregnancy-related risk factors

NHANES surveys for 1999–2006 included two self-reported pregnancy-related factors associated with CVD: (1) history of a LBW infant and (2) history of breastfeeding. A LBW infant was defined as delivery of any infant weighing less than 2500 g at birth. A history of breastfeeding was defined as breastfeeding of an infant for 1 month or more.

### Outcome measures

The primary outcome was CVD death, defined as leading cause of death due to disease of the heart (ICD codes I00–I09, I11, I13 and I20-I51) or leading cause of death due to cerebrovascular disease (ICD codes I60-I69) [25]. NHANES survey data was linked to the public-use National Death Index (NDI) in the 2015 Linked Mortality File (LMF), in which mortality status and follow-up time for NHANES survey participants was ascertained through probabilistic record matching with the NDI [25]. For NHANES, the public-use LMF organizes the specific cause-of-death codes into nine leading cause of death categories, including the two leading cause of death categories used in this study. Certain contributing causes of death are coded in multiple causes of death codes, though only hypertension and diabetes are available in the public-use LMF and were used for the secondary outcome described below.

NHANES does not include data on incident non-fatal myocardial infarction or stroke, two outcomes predicted by the ACC/AHA PCE risk model. We created a proxy variable, termed CVD deaths + surrogate CVD. Surrogate CVD was defined as non-CVD deaths with diabetes or hypertension identified as a contributing a cause of death. We assumed that participants for whom hypertension or diabetes was a contributing cause of death may have been more likely to have experienced a non-fatal MI or stroke.

### CVD prediction model and other control variables

The ACC/AHA PCE predicts the 10-year risk of first CVD outcome using traditional risk factors of age (years), total cholesterol (mg/dL), high-density lipoprotein cholesterol (mg/dL), treated or untreated systolic blood pressure (mmHg), diabetes, and current smoking status [3]. Four equations were developed to estimate risk by sex and by race (Black or White; other non-White participants were classified as White by the PCE) [3]. The average of up to 4 readings of systolic blood pressure was computed according to physician protocol [26]. Treatment of hypertension was self-reported and medications were verified by study staff. Diabetes was defined as (1) self-reported diagnosis, (2) use of oral anti-glycemic agent or insulin, or (3) Hemoglobin A1c level ≥ 6.5% [27]. Current smoker was defined as self-reported tobacco use of at least 100 cigarettes in one’s lifetime and a smoking frequency of every day or some days, consistent with prior classification in NHANES [28].

Models were adjusted for socioeconomic factors (marital status, education level), pregnancy variables (parity, age at first live birth), and health factors (body mass index (BMI), physical activity, diet). Diet quality was measured by the Healthy Eating Index 2015, which was constructed according to NIH guidance using NHANES dietary data, USDA Food Patterns Equivalents Database, USDA's Center for Nutrition Policy, and Promotion MyPyramid Equivalents Databases for Whole Fruit and Fruit Juice [29–31]. Weekly minutes of physical activity meeting was created according to NHANES protocols [32, 33].

### Statistical analyses

Bivariate analysis was performed using Student’s t-test for continuous variables and Chi-square test for categorical variables. Multiple imputation using Bayesian method was performed for the missing cases in the PCE risk score variables and control variables using normal linear model for continuous variables and logistic regression for binary variables with 25 complete data sets. Missing values of LBW infant and breastfeeding were not imputed.

Cox proportional hazards models were used to evaluate the association between pregnancy-related factors and CVD outcomes, adjusting for PCE risk score and control variables (marital status, education level, BMI, parity, age at first birth, weekly hours of physical activity, Healthy Eating Index 2015). Schoenfeld residuals were used to verify the proportional hazards assumption. Follow-up time was calculated from the date of the NHANES medical exam to the date of participant death due to CVD or the end of the follow-up period (December 31, 2015), whichever came first. Deaths from causes other than eligible CVD outcomes were also censored. Additionally, survival analysis using competing risks was performed, where death from causes other than CVD were treated as competing risks [34]. Follow-up time in the competing risks analysis was calculated from the date of the medical exam to CVD death or the end of the follow-up period for those with a non-CVD death or no death. All statistical analyses were performed in R version 3.6.1, accounting for complex survey design in NHANES by utilizing appropriate sample weights, primary sampling units and strata [33]. Statistical significance was defined as *p* < 0.05.

We planned to add each pregnancy-related factor to the PCE, following guidance from the AHA on evaluation of novel cardiovascular biomarkers [35], re-estimating risk equations with the pregnancy-related factor, comparing model discrimination and calibration, and assessing net reclassification improvement. Due to the small number of CVD events, we could not perform these analyses.

## Results

A total of 3758 of women ages 40 to 79 with a pregnancy history and no pre-existing CVD were identified, representing an estimated 25,964,134 women in the US. Seventy-five percent of women were White, 10 percent Black, and the remaining 15 percent Mexican American, other Hispanic, or other/mixed race (Table 1). The mean gravidity and parity were 3.5 and 2.7, respectively. Of the sample, 479 (12.1%) had a history of a LBW infant and 1926 (53.1%) had breastfed an infant for greater than 1 month. The mean 10-year risk of a CVD event estimated by the PCE for the sample was 6.7%; 27.3% had a 10-year risk of CVD > 7.5% (intermediate and high risk). Less than 5% of participants were missing data on control variables and PCE predictor variables (LBW infant 4.5% and breastfeeding 3.8%). Mean follow-up time was 12.1 years. Twelve percent of women were deceased at follow-up (n = 588). Eighty-seven women (1.5%) had a CVD death and 190 (3.2%) had a CVD death + surrogate CVD outcome (Table 2).

**Table 1:** Descriptive Statistics of Women with Pregnancy History in the National Health and Nutrition Examination Survey, 1999–2006. (n = 3758)

	Total (n = 3758)	LBW infant (n = 479)	No LBW infant (n = 3111)	*p* value	Breastfeeding (n = 1926)	No Breastfeeding (n = 1689)	*p* value
**Demographic variables**							
Age, years (n = 3785)	55.3 ± 0.3	55.9 ± 0.6	55.4 ± 0.3	0.55	54.3 ± 0.4	56.8 ± 0.4	< 0.01
Race, %				0.58			
Mexican American (n = 866)	4.9 ± 0.6	5.6 ± 1.0	4.9 ± 0.6		6.1 ± 0.8	4.0 ± 0.6	< 0.01
Other Hispanic (n = 149)	4.5 ± 0.8	2.5 ± 1.0	4.8 ± 0.9		5.8 ± 1.2	3.1 ± 0.7	
Non-Hispanic White (n = 1856)	75.8 ± 1.6	73.0 ± 2.3	75.9 ± 1.7		75.5 ± 1.8	75.4 ± 1.8	
Non-Hispanic Black (n = 762)	10.4 ± 1.1	14.0 ± 1.7	9.9 ± 1.1		7.4 ± 0.8	13.9 ± 1.6	
Other Race-Including Multi-Racial (n = 125)	4.4 ± 0.5	4.8 ± 1.2	4.4 ± 0.6		5.3 ± 0.9	3.6 ± 0.6	
Education, %				< 0.01			0.15
Less than high school (n = 1173)	18.0 ± 0.8	25.9 ± 2.5	17.2 ± 0.9		17.1 ± 1.2	19.9 ± 1.3	
High school or higher (n = 2582)	82.0 ± 0.8	74.1 ± 2.5	82.8 ± 0.9		82.9 ± 1.2	80.1 ± 1.3	
Marital Status (%)				0.07			0.02
Not married (n = 1515)	35.3 ± 1.2	35.3 ± 3.3	34.1 ± 1.1		32.1 ± 1.5	36.9 ± 1.6	
Married (n = 2135)	64.7 ± 1.2	64.7 ± 3.3	65.9 ± 1.1		67.9 ± 1.5	63.1 ± 1.6	
**Health factors**							
Body Mass Index, kg/cm2 (n = 3725)	28.7 ± 0.2	28.1 ± 0.4	28.8 ± 0.2	0.18	28.5 ± 0.2	29 ± 0.2	0.09
Physical Activity, Minutes/Week (n = 3784)	369.5 ± 14.9	342.2 ± 35.8	367.4 ± 15.5	0.48	406.4 ± 22.3	316.3 ± 18.1	< 0.01
HEI score (n = 3650)	51.0 ± 0.4	50.2 ± 0.8	50.8 ± 0.4	0.51	52.2 ± 0.4	49.0 ± 0.5	< 0.01
**Ob/Gyn Variables**							
Age of first menstrual period (n = 3692)	12.8 ± 0.0	12.8 ± 0.1	12.8 ± 0.1	0.56	12.8 ± 0.1	12.7 ± 0.1	0.54
Age at first live birth (n = 3638)	23.1 ± 0.2	22.2 ± 0.3	23.2 ± 0.2	< 0.01	23.8 ± 0.2	22.3 ± 0.2	< 0.01
Parity (%)				0.12			< 0.01
1 live birth (n = 616)	18.5 ± 0.9	11.0 ± 2.2	15.4 ± 0.9		11.8 ± 1.2	18.5 ± 1.1	
> 1 live birth (n = 3126)	81.5 ± 0.9	89.0 ± 2.2	84.6 ± 0.9		88.2 ± 1.2	81.5 ± 1.1	
**Traditional Risk Factors used in PCE**							
Treated systolic blood pressure (n = 1182)	135.6 ± 1.0	140.1 ± 1.7	135.1 ± 1.0	0.02	133.7 ± 1.1	137.5 ± 1.2	0.01
Untreated systolic blood pressure (n = 2522)	124 ± 0.6	126.2 ± 1.3	123.9 ± 0.6	0.12	122.2 ± 0.7	126.8 ± 0.8	< 0.01
Total Cholesterol (n = 3618)	212.6 ± 1.0	212.4 ± 2.8	212.6 ± 1.1	0.92	210 ± 1.4	215.6 ± 1.5	0.64
HDL Cholesterol (n = 3619)	59.4 ± 0.4	60.6 ± 1.0	59.1 ± 0.5	0.17	59.1 ± 0.5	59.5 ± 0.6	0.09
Current Smoking, % (n = 649)	18.3 ± 0.9	25.4 ± 3.1	17.3 ± 1.0	< 0.01	13.3 ± 1.1	24.0 ± 1.2	< 0.01
Diabetes, % (n = 556)	9.8 ± 0.6	8.2 ± 1.2	10.3 ± 0.6	0.13	9.0 ± 0.7	11.2 ± 0.9	0.05
10-year estimated CVD risk, % (n = 3487)	6.7 ± 0.2	7.3 ± 0.5	6.7 ± 0.2	0.26	5.8 ± 0.3	8.1 ± 0.3	< 0.01
PCE risk categories, %				0.04			< 0.01
Low (< 5%) (n = 1860)	62.6 ± 1.3	55.6 ± 3.1	62.8 ± 1.5		68.9 ± 1.7	53.6 ± 2.1	
Borderline low (5- < 7.5%) (n = 361)	10.1 ± 0.9	14.1 ± 2.0	9.6 ± 0.9		8.6 ± 1.1	12.0 ± 1.0	
Intermediate (7.5- < 20% (n = 828)	18.9 ± 0.8	22.3 ± 2.6	19.1 ± 0.9		15.7 ± 1.0	24.0 ± 1.4	
High (> = 20%) (n = 438)	8.4 ± 0.5	8.1 ± 1.2	8.5 ± 0.6		6.9 ± 0.7	10.5 ± 0.8)	

LBW infant defined as delivery of any infant weighting less than 5 ½ pounds at birth. Breastfeeding defined as breastfeeding any infant for 1 month or more. CVD deaths defined as deaths with heart disease (ICD I00–I09, I11, I13 and I20-I51) or cerebrovascular disease (ICD I60-I69) identified as the leading cause of death. CVD deaths + surrogate CVD defined as CVD deaths and deaths with diabetes or hypertension identified as a secondary cause of death

Percent data are weighted by the sampling weights % ± standard error

The groups of women with and without a pregnancy-related risk factor do not add up to the column total due to missing data for that risk factor. (LBW missing, n = 168; Breastfeeding missing, n = 143)

CVD, cardiovascular disease; HEI, Healthy Eating Index; LBW, low birthweight infant; Ob/Gyn, obstetrics/gynecology

**Table 2:** Bivariate Statistics of Women with Pregnancy History in the National Health and Nutrition Examination Survey, 1999–2006. (n = 3758)

	Total (n = 3758)	LBW infant (n = 479)	No LBW infant (n = 3111)	*p* value	Breastfeeding (n = 1926)	No Breastfeeding (n = 1689)	*p* value
Outcomes							
CVD deaths, n (%)	87 (1.5)	9 (1.7)	72 (1.5)	0.82	44 (1.3)	40 (1.8)	0.28
CVD deaths + surrogate CVD, n (%)	190 (3.2)	28 (4.3)	154 (3.1)	0.20	89 (2.8)	96 (3.9)	0.09

LBW infant defined as delivery of any infant weighting less than 5 ½ pounds at birth. Breastfeeding defined as breastfeeding any infant for 1 month or more. CVD deaths defined as death with heart disease (I00–I09, I11, I13 and I20-I51) or cerebrovascular disease (I60-I69) identified as the leading cause of death. CVD deaths + surrogate CVD defined as CVD deaths and deaths with diabetes or hypertension as a secondary cause of death

Percent data are weighted by the sampling weights % ± standard error

The groups of women with and without a pregnancy-related risk factor do not add up to the column total due to missing data for that risk factor. (LBW missing, n = 168; Breastfeeding missing, n = 143)

CVD, cardiovascular disease; LBW, low birthweight

In the bivariate analysis, women with a LBW infant were more likely to be Black (14% vs 10%, *p* = 0.06), have higher blood pressure (treated or untreated), be current smokers (25% vs 15%, *p* < 0.01), and have a higher mean 10-year risk of CVD (7.3% vs 6.7%, *p* = 0.26) compared to women without a LBW infant (Table 1). Women with a LBW infant also had lower levels of moderate to vigorous exercise and lower diet quality scores. Similar CVD death rates were observed in the LBW group compared with the no LBW group (9 (1.7%) vs 72 (1.5%), *p* = 0.82); including CVD surrogate outcome yielded similar results (LBW infant 4.3% vs. no LBW infant 3.1%, *p* = 0.20) (Table 2).

Breastfeeding women had more favorable cardiovascular risk factor profiles compared to non-breastfeeding women, including lower systolic blood pressure (treated or untreated), lower smoking (13.3% vs. 24.0%, *p* ≤ 0.01), diabetes (9.0% vs. 11.2%, *p* = 0.05), and 10-year CVD risk (5.8% vs 8.1%, *p* < 0.01) (Table 1). Women who breastfed were more likely to report healthy behaviors (diet quality scores: 52.2 vs 49.0, *p* < 0.01; weekly minutes of moderate to vigorous exercise: 406.4 vs 316.3, *p* < 0.01). There was a lower proportion of CVD deaths (1.3% vs. 1.8%, *p* = 0.28) and CVD deaths + surrogate outcomes (2.8% vs. 3.9%, *p* = 0.09) in the breastfeeding group compared with women who did not breastfeed (Table 2).

Table 3 shows the cause-specific survival model for LBW infant with the outcome of CVD death, where the hazard ratio approached the null (HR 0.98; 95% CI 0.47─2.05; *p* = 0.96). For the competing risk model, the hazard ratio was less than 1, though also with a wide confidence interval and non-significant results (HR 0.86, 95% CI 0.41─1.81, *p* = 0.69). For the outcome of CVD deaths + surrogate CVD, the results remained statistically non-significant. (Cause-specific model: HR 1.29; 95% CI 0.74─2.25; *p* = 0.38; competing risks model: HR 1.10; 95% CI 0.65─1.88; *p* = 0.71).

**Table 3:** Survival analysis for low birthweight as a predictor of cardiovascular death and surrogate outcomes for women with pregnancy history in the National Health and Nutrition Examination Survey, 1999–2006

	CVD death				CVD deaths + surrogate CVD			
	Unadjusted		Adjusted		Unadjusted		Adjusted	
	HR (95% CI)	*p* value	HR (95% CI)	*p* value	HR (95% CI)	*p* value	HR (95% CI)	*p* value
Cause-specific survival model	1.11 (0.52–2.38)	0.78	0.98 (0.47─2.05)	0.96	1.43 (0.86–2.37)	0.17	1.29 (0.74─2.25)	0.38
Competing risks survival model	1.07 (0.51–2.26)	0.86	0.86 (0.41─1.81)	0.69	1.27 (0.77–2.10)	0.34	1.10 (0.65─1.88)	0.71

Cause-specific models were censored at the time of death of non-CVD or end of follow-up period. Competing risks models were censored at the end of follow-up period regardless of non-CVD death. Models were adjusted for marital status, education level, body mass index, parity, age at first birth, weekly hours of physical activity, Healthy Eating Index 2015, and Pooled Cohort Equation 10-year estimated CVD risk

CI, confidence interval; CVD, cardiovascular disease; HR, hazard ratio

Survival models for breastfeeding showed a consistent reduction in CVD for outcomes of CVD death and CVD death + surrogate CVD, though estimates were not statistically significant. (Table 4) In the cause-specific survival model, breastfeeding was associated with a 24 percent reduction in risk of CVD deaths (HR 0.76; 95% CI 0.45─1.27, *p* = 0.30) and 33 percent reduction in risk of CVD deaths + surrogate CVD (HR 0.77; 95% CI 0.52─1.14, *p* = 0.19). Similar results were observed for the competing risks models (CVD deaths: HR 0.79; 95% CI 0.48─1.33, *p* = 0.38 and CVD deaths + surrogate CVD: HR 0.77; 95% CI 0.52─1.14, *p* = 0.20).

**Table 4:** Survival analysis for breastfeeding as a predictor of cardiovascular death and surrogate outcomes for women with pregnancy history in the National Health and Nutrition Examination Survey, 1999–2006

	CVD Death				CVD deaths + surrogate CVD			
	Unadjusted		Adjusted		Unadjusted		Adjusted	
	HR (95% CI)	*p* value	HR (95% CI)	*p* value	HR (95% CI)	*p* value	HR (95% CI)	*p* value
Cause-specific survival model	0.75 (0.44–1.27)	0.28	0.76 (0.45─1.27)	0.30	0.72 (0.48–1.06)	0.10	0.77 (0.52─1.14)	0.19
Competing risks survival model	0.76 (0.45–1.30)	0.32	0.79 (0.48─1.33)	0.38	0.77 (0.52–1.12)	0.17	0.77 (0.52─1.14)	0.20

Cause-specific models were censored at the time of death of non-CVD or end of follow-up period. Competing risks models were censored at the end of follow-up period regardless of non-CVD death. Models were adjusted for marital status, education level, body mass index, parity, age at first birth, weekly hours of physical activity, Healthy Eating Index 2015, and Pooled Cohort Equation 10-year estimated CVD risk

CI, confidence interval; CVD, cardiovascular disease; HR, hazard ratios

We intended to assess prediction improvement by adding each pregnancy-related factor to the PCE; however, we were unable to due to the small number of CVD outcomes.

## Discussion

Our study examining the association of pregnancy-related factors and CVD outcomes suggests that a history of breastfeeding was associated with a greater than 20 percent reduction in CVD deaths, adjusting for traditional risk factors in the PCE and behavioral factors. Including surrogate CVD outcomes increased risk reduction to 33 percent, with results approaching statistical significance. The association between LBW infants and CVD risk was less certain, in part due to the low number of available CVD outcomes among women with a LBW infant. Low number of CVD events also precluded assessing if including pregnancy-related factors in the PCE score led to reclassification of CVD risk category and improved CVD risk stratification. Despite limitations, our results make important contributions to the literature on pregnancy-related CVD risk and have implications for future research and clinical practice.

Lactation has been associated with favorable effects on maternal glucose homeostasis, lipid profiles, and weight loss [36, 37], and there is a growing recognition that the maternal benefits of breastfeeding can extend beyond the postpartum period [21]. Prior prospective studies including the US Nurse’s Study [38], the Women’s Health Initiative [39, 40], and non-US cohorts [20] have reported similar inverse associations between breastfeeding and CVD outcomes, ranging from the CVD outcomes used in the PCE to revascularization and self-reported anginal symptoms. Unlike prior studies, we controlled for the complete set of traditional risk factors in the PCE recommended by leading professional organizations for primary prevention of CVD [3–5, 22]. Breastfeeding status can be influenced by socioeconomic status and environmental factors, for which we included control variables available in NHANES (education and marital status). Some investigators have argued that the association of breastfeeding and CVD may be explained by reverse causality—pre-pregnancy risk factors predict CVD rather than breastfeeding itself [41]. The present dataset did not contain pre-pregnancy data, though our models did control for the healthier behaviors of breastfeeding women (physical activity and diet quality), and our results continued to suggest a reduction in CVD outcomes.

The association of a LBW infant and CVD risk is limited by the small number of CVD outcomes in NHANES and may also be related to the overall lower prevalence of this condition. Only 9 women giving birth to a LBW infant had a CVD death. The inverse relationship between LBW infant and CVD death cannot be interpreted given the wide 95% confidence interval that included 1. Including surrogate CVD outcomes, the number of outcomes in the exposed group increased and the association between LBW infant and CVD flipped to the expected direction, though again the confidence interval included the null. Evidence on the association of low birthweight infants and CVD events is limited and mixed. In cohort studies conducted using Scottish registry data, birth of a LBW infant was associated with ischemic heart disease (HR 4.3, 95% CI 2.9–6.2) though not ischemic stroke [42, 43]. Literature of small for gestational age (SGA) infant, defined as birthweight below the 10^th^ percentile and a subset of LBW infants, is similarly mixed. In a systematic review examining the association of giving birth to an SGA infant and CVD, authors reported hazard ratios ranging from 1.09 to 2.5, though heterogenous definitions of SGA and CVD outcomes among the studies precluded more robust evidence synthesis. Further research is required to assess if our results represent a type 2 error or a true null association between giving birth to an LBW infant and CVD outcomes.

A small number of studies have examined the contribution of pregnancy-related factors to a risk prediction model, as this study attempted to do [44]. The main limitations of this research base include limited CVD outcomes, pregnancy-related factors, and racial and ethnic diversity [39, 45–47]. Our study had similar limitations. CVD death is the only eligible outcome in NHANES. We attempted to mitigate this limitation using a CVD surrogate outcome where deaths with a secondary cause of diabetes or hypertension were considered non-fatal CVD events, though we were unable to confirm underlying CVD events in NHANES and there is a risk of misclassification. Likewise, we could only evaluate two pregnancy-related factors. In later years, NHANES included gestational diabetes in their survey; however, < 10 CVD outcomes were identified in 2015 LMF for NHANES survey years 2007–2014, precluding further analysis. Recall bias may be considered for LBW given self-report, though a study of maternal recall showed that sensitivity of recall varied according to specific pregnancy-related factor, with recall of LBW showing no consistent pattern of bias compared with medical record documentation [48]. Longer periods of breastfeeding have been associated with improved outcomes [49]; however, NHANES did not stratify breastfeeding duration beyond 1 month. Risk estimation in non-White populations is subject to ongoing validity concerns because the PCE were derived from primarily white cohorts and the PCE tends to overestimate CVD risk in more diverse, contemporary cohorts [50, 51]. Beyond data limitations, conceptual limitations of risk prediction for younger women, as represented by this sample, remain. Younger women intrinsically have a lower estimated 10-year CVD risk because age is such a strong predictor in the PCE [52]. Biomarkers such as apoB and lifetime risk prediction models may be able to target people at younger ages, though research in these fields is ongoing [53, 54].

Limitations notwithstanding, our study has several strengths and implications for clinical practice. We examined a nationally representative sample that oversamples Black and Hispanic individuals. We used a CVD risk prediction model that is recommended by clinical societies, used in clinical practice, and can be (or is) integrated into electronic medical records and clinical workflows by non-physician staff. Similarly incorporating screening for pregnancy-related factors could augment early identification of high-risk women, particularly younger women and women in the peripartum period. Including pregnancy-related factors into CVD risk assessment offers strategies for integrating prevention across disciplines of Ob/Gyn and internal medicine. For example, CVD screening in Ob/Gyn offices could prompt a warm hand-off to primary care for longer-term follow-up as recently recommended by a joint statement between the AHA and ACOG, or medicine referrals could be initiated while patients are still inpatients postpartum [55, 56]. Preconception counseling also plays a role in promoting breastfeeding for maternal as well as infant benefit.

Future research to deepen the understanding of the relationship between pregnancy-related factors and CVD risk will require more robust data on pregnancy complications, documentation of CVD outcomes, and diverse cohorts. Quantifying the additional cardiovascular risk of pregnancy-related factors remains a necessary step to guide clinicians in how to best manage potential cardiovascular risk factors independent of traditional CVD risk factors. Interventions studies are needed to assess whether aggressive prevention interventions earlier in a women’s life when she is diagnosed with a pregnancy complication results in fewer CVD outcomes. 

## Conclusion

Women with a history of breastfeeding in a nationally representative sample may have a reduced risk of CVD outcomes. There was no clear association between LBW infant and CVD outcomes. The evaluation of pregnancy-related conditions holds promise to improve CVD risk assessment and outcomes in women with a history of pregnancy.

## Data Availability

The data that support the findings of this study are publicly available in the National Center for Health Statistics, National Health and Nutrition Examination Survey (https://wwwn.cdc.gov/nchs/nhanes/Default.aspx).

## Abbreviations

ACC: American College of Cardiology
AHA: American Heart Association
BMI: Body mass index
CVD: Cardiovascular disease
LBW: Low birthweight infant
NHANES: National Health and Nutrition Examination Survey
PCE: Pooled Cohort Equation
